# Identifying differences in the experience of (in)authenticity: a latent class analysis approach

**DOI:** 10.3389/fpsyg.2014.00770

**Published:** 2014-07-15

**Authors:** Alison P. Lenton, Letitia Slabu, Martin Bruder, Constantine Sedikides

**Affiliations:** ^1^Department of Psychology, University of EdinburghEdinburgh, UK; ^2^Department of Psychology, Middlesex UniversityLondon, UK; ^3^Zukunftskolleg and Department of Psychology, University of KonstanzKonstanz, Germany; ^4^Centre for Research on Self and Identity, School of Psychology, University of SouthamptonSouthampton, UK

**Keywords:** authenticity, inauthenticity, latent class analysis, self, culture

## Abstract

Generally, psychologists consider state authenticity – that is, the subjective sense of being one’s true self – to be a unitary and unidimensional construct, such that (a) the phenomenological experience of authenticity is thought to be similar no matter its trigger, and (b) inauthenticity is thought to be simply the opposing pole (on the same underlying construct) of authenticity. Using latent class analysis, we put this conceptualization to a test. In order to avoid over-reliance on a Western conceptualization of authenticity, we used a cross-cultural sample (*N* = 543), comprising participants from Western, South-Asian, East-Asian, and South-East Asian cultures. Participants provided either a narrative in which the described when they felt most like being themselves or one in which they described when they felt least like being themselves. The analysis identified six distinct classes of experiences: two authenticity classes (“everyday” and “extraordinary”), three inauthenticity classes (“self-conscious,” “deflated,” and “extraordinary”), and a class representing convergence between authenticity and inauthenticity. The classes were phenomenologically distinct, especially with respect to negative affect, private and public self-consciousness, and self-esteem. Furthermore, relatively more interdependent cultures were less likely to report experiences of extraordinary (in)authenticity than relatively more independent cultures. Understanding the many facets of (in)authenticity may enable researchers to connect different findings and explain why the attainment of authenticity can be difficult.

## INTRODUCTION

It is the chief point of happiness that a man is willing to be what he is.

Desiderius Erasmus (The Praise of Folly, 1511/1668).

Psychological scientists have defined authenticity in a variety of ways, with most definitions emphasizing the need for a high degree of correspondence between a person’s behavior and her/his “true self” (Harter, 2002; Kernis and Goldman, 2006; Wood et al., 2008). This definition stems from a dispositional view of authenticity, which has been the predominant narrative in the empirical literature to date. More recently, however, researchers have viewed authenticity through a situational or “state” lens (Heppner et al., 2008; Fleeson and Wilt, 2010; Gino et al., 2010; Lenton et al., 2013a,b). We concur with Fridhandler (1986) that “if a person is in a state he or she must be able to feel it” (p. 170). This is especially true for state authenticity, which is thought to act as a signal (or warning system, in the case of state inauthenticity) of one’s current degree of self-coherence and self-integration (Erickson, 1995; Sheldon et al., 1997). Stated otherwise, state authenticity (and its counterpart, inauthenticity) is a phenomenological experience.

Authenticity is also emerging as a societal imperative. Whether it comes from parents, politicians, celebrities, Web gurus, or college admissions advisers, the advice is similar: “just be yourself” (Rosenbloom, 2011). This advice echoes the large number of websites addressing “how to be yourself” (*Google* search: 60.2 million hits as of May 15, 2014) and the growing number of self-help books on being “authentic” (1686 books on *Amazon.com* as of May 15, 2014). In everyday life, people report experiencing authenticity once or twice each week and most are strongly motivated to attain authenticity and avoid inauthenticity (Lenton et al., 2013a). Because so many people are seeking authenticity, it is clearly important to improve our understanding of how it can (and cannot) be attained.

Amongst social psychologists, *authenticity* refers to the personal sense that one is “real” or “true” (Harter, 2001). Prior research, most of which has been conducted in Western cultures, indicates that dispositional (trait) authenticity is associated with increased positive affect, decreased negative affect, greater self-esteem, more life-satisfaction (Goldman and Kernis, 2002), higher well-being, and lower stress (Wood et al., 2008). State authenticity has similarly positive experiential correlates: greater positive affect, less negative affect, higher well-being, lower public self-consciousness, increased self-esteem, more need satisfaction, and greater alignment with the ideal self (Heppner et al., 2008; Lenton et al., 2013a; Robinson et al., 2013). It would appear that authenticity is a uniformly positive, contentment-inducing experience, whereas inauthenticity – authenticity’s counterpart – is a uniformly negative, anxiety-provoking experience. These findings resonate with what we regard as the dominant conceptualization of authenticity: Authenticity is an unidimensional construct, with inauthenticity simply denoting the opposing end of the “authenticity spectrum.” But is this necessarily so? The present investigation aimed to assess relevant evidence regarding state authenticity using a large, cross-cultural sample.

Different strands of research suggest that not all instances of state (in)authenticity may feel the same, as triggering circumstances vary. For example, people feel inauthentic if they are required to manage emotional expressions for their job (e.g., “service with a smile”; Hochschild, 1983) or when wearing counterfeit sunglasses (Gino et al., 2010). Also, whereas the experience of authenticity is primarily associated with fun and familiarity, some individuals report authenticity even when facing personal difficulties. Similarly, although the experience of inauthenticity is largely associated with feeling judged by others, some individuals report inauthenticity when being helpful (Lenton et al., 2013a). The phenomenological experiences of (in)authenticity, then, may be more complex than mean scale scores (e.g., average positive affect, average public self-consciousness) convey. Stated otherwise, the “inauthenticity” felt when regularly falsifying one’s emotional expressions may be qualitatively – not just quantitatively – different from that felt when briefly donning counterfeit sunglasses.

To illustrate this idea further, self-awareness has been theorized both to increase (Kernis and Goldman, 2006; Wood et al., 2008) and decrease (Turner and Billings, 1991; Leary et al., 2006) authenticity. Kernis and Goldman (2006) suggested that the authentic individual is consciously aware of her/his multi-faceted (yet contradictory) nature and this awareness facilitates a cohesive self. In contrast, Leary et al. (2006) argued that a person can behave more naturally and make better progress toward her/his goals when self-awareness is lowered. Yet, in our earlier work investigating what state authenticity feels like, the average level of private self-consciousness was at the scale’s mid-point (Lenton et al., 2013a), apparently supporting neither hypothesis. In that work, however, we examined (in)authenticity as if it were a unitary construct. Thus, this result may indicate evidence for both hypotheses, if there are (at least) two distinct types of authenticity: one low, one high in private self-consciousness.

To examine such possibilities, we conducted a latent class analysis (LCA) on previously collected cross-cultural data. LCA is a powerful tool for revealing hidden data patterns, as it “aim(s) to uncover unobserved heterogeneity in a population and to find substantively meaningful groups [..] that are similar in their responses” (Nylund et al., 2007, p. 536). We address the distinction between group and construct heterogeneity in further detail in the section “Discussion.” Researchers have speculated (Goldman and Kernis, 2002) that the sense of authenticity facilitates self-coherence and well-being. But what if (in)authenticity is a multi-faceted experience? If so, not all instances of (in)authenticity may have the same benefits for well-being and the self. Additionally, if subtype experiences exist, LCA may identify critical experiential aspects that are common to all forms of authenticity or inauthenticity. For example, perhaps high positive affect pervades all instances of authenticity, but high need satisfaction does not. This would suggest that positive affect is a necessary feature of the authenticity experience, whereas need satisfaction is not.

Furthermore, what if cultures differ in terms of which facets they experience? It is possible that benefits associated with authenticity – such as higher self-esteem, higher positive affect, and lower negative affect (Goldman and Kernis, 2002), as well as decreased stress (Wood et al., 2008) – are restricted to cultures valuing individualism (Robinson et al., 2013). Western cultures promote a relatively independent view of the self, whereas Eastern cultures promote a relatively interdependent view of the self (Markus and Kitayama, 1991). Authenticity, which is generally conceptualized as the sense of a distinct true or real self, seems more closely related to the Western view of the self, suggesting, perhaps, that the experience of (and search for) authenticity is a by-product of Western ideals. Alternatively, authenticity may actually relate to one’s internal cultural norms (Sherman et al., 2012). For example, East Asians (e.g., Chinese, Japanese) perceive personality as a malleable entity, whereas Westerners (e.g., Americans, British) perceive it as a fixed entity (Chiu et al., 1997). It would follow that East Asians may feel more authentic when adapting to and Westerners when resisting social pressures. In other words, individuals from both cultures may experience authenticity and reap its benefits, but perhaps under different circumstances.

Few studies of authenticity have adopted a cross-cultural perspective (Robinson et al., 2013; Slabu et al., in press), let alone been conducted outside of Western cultures (but see Ito and Kodama, 2007, 2008). Robinson et al. (2013) did not assess directly the phenomenology of authenticity, but they discovered that the (trait) authenticity–well-being relation was similarly strong across three cultures (USA, England, Russia). In our cross-cultural (USA, India, China, Singapore) investigation, we found that culture moderated the subjective experience of state (in)authenticity (Slabu et al., in press). For example, Indian participants were more likely than their counterparts to report experiencing higher private self-consciousness when feeling authentic, but less likely to report experiencing increased self-esteem in the same situation. Again, however, in the Slabu et al. (in press) investigation we assumed authenticity to be a unitary experiential construct; that is, we averaged across a wide array of experiences to assess how authenticity and inauthenticity differ from one another. If different cultures experience qualitatively distinct types of (in)authenticity, then the authenticity–well-being relation uncovered by Robinson et al. (2013) likely has a different foundation across cultures (i.e., authenticity facilitates well-being for different reasons). Furthermore, it would suggest that cultures vary in their conceptualizations of what it means to “feel like my true self.” Thus, the current study also adds to the emerging literature examining if and how authenticity differs across cultures.

## MATERIALS AND METHODS

### PARTICIPANTS

We recruited participants from one of three sources: (a) University students in the UK, Singapore, and China (the latter two via local contacts); (b) persons visiting our own or other websites listing psychological studies; and (c) persons registered with Amazon’s Mechanical Turk (MTurk), a global website that offers online tasks for pay (in our study: $3–$4, *n* = 325). Individuals from more than 60 countries responded, but for most countries the number of participants was insufficient for an LCA analysis (e.g., 56% of the countries had only 1 response). Additionally, we excluded participants who indicated that they were resident in a country other than their country of origin for more than 5 years, because immersion into the host culture likely dilutes the original cultural socialization (Masgoret and Ward, 2006). The final data consisted of 523 individuals from two samples comprising four cultural groups: (a) Western, English-speaking countries (*n* = 242; in descending frequency: United States, United Kingdom, Canada, New Zealand, Australia, Ireland); (b) South-Asian countries (*n* = 82; India, Pakistan); (c) East-Asian countries (*n* = 105; China, Japan); (d) South-East Asian countries (*n* = 94; Singapore, Philippines, Indonesia, Malaysia, Thailand). Elsewhere we have reported analyses using data from the same participant samples (Lenton et al., 2013a; Slabu et al., in press). Here, we combined and analyzed those data (along with data previously unreported) using LCA.

#### Sample 1

This sample comprised 84 volunteers. It included 64 female and 20 male participants (*M*_age_ = 30.90 years, SD_age_ = 12.79), nearly all from Western, English-speaking countries (one from East-Asia).

#### Sample 2

This sample comprised 439 volunteers. It included 272 female and 167 male participants (*M*_age_ = 26.44 years, SD_age_ = 8.76 years). Given that the cultural make-up of this sample was diverse, we asked non-native English speakers to rate their English proficiency in reading and writing on a 5-point scale (1 = *not at all fluent*, 5 = *perfectly fluent*). Most participants from Western (96.2%) and South-East Asian (64.9%) countries were native English speakers. English was a native language for approximately one-quarter (25.6%) of South-Asian and 1.0% of East-Asian participants. Of non-native English speakers, 100% of Westerners, 97.0% of South-East Asians, 100% of South-Asians, and 92.2% of East-Asians reported having a *fair* or better English proficiency (at least 3 on the 5-point scale reported above).

### PROCEDURE

Participants completed an online survey (in English) and they were randomly assigned to one of two conditions that only differed with respect to the memories to be narrated. In the first condition, participants were instructed to describe an event during which “you felt ***most*** like your *true* or *real self* ” (most-me condition), whereas, in the second condition, they were asked to describe an event during which “you felt ***least*** like your *true* or *real self* ” (least-me condition). All participants then rated that event (1 = *strongly disagree*, 7 = *strongly agree*) on: (a) positive and negative affect (Kercher, 1992, short-form PANAS for Sample 1; Thompson, 2007, international short-form PANAS for Sample 2); (b) self-esteem (Rosenberg, 1965); (c) private (nine items) and public (three items) self-consciousness (Fenigstein et al., 1975); (d) ideal-self overlap (10 attributes from Self-Attributes Questionnaire – e.g., attractiveness, humor; Pelham and Swann, 1989); and (e) need satisfaction (one item per each of 10 psychological needs – e.g., autonomy, competence; Sheldon et al., 2001). Instructions were adapted so that participants rated their psychological state in that situation (e.g., state self-esteem) rather than generally (e.g., trait self-esteem). All measures were reliable (Cronbach’s αs ≥ 0.83).

## RESULTS

### DATA PREPARATION

Given that scale usage can depend on culture (e.g., acquiescence, extreme responding; Van Vaerenbergh and Thomas, 2013), we controlled for culture-based response biases. This is especially important when employing analytic methods that rely upon correlations: LCA partly works to identify classes by assessing correlations among indicators (Magidson and Vermunt, 2004). If response biases are not controlled, conclusions may be invalid (Fischer and Milfont, 2010; Van Vaerenbergh and Thomas, 2013). Specifically, we did not want classes to reflect varieties of response styles but, rather, varieties of authenticity or inauthenticity experiences.

Regression analyses showed that culture (Western, South Asian, East Asian, South-East Asian) had significant effects on extreme response style, *F*(3,519) = 16.85, *p*= 0.001, *R*^2^ = 0.09, and acquiescent response style, *F*(3,519) = 21.86, *p*= 0.001, *R*^2^ = 0.11 (analytic details available upon request). Accordingly, we standardized the indicators within each culture (Fischer and Milfont, 2010) by subtracting their culture’s mean across all items from participants’ raw scale scores for each indicator and then dividing by their culture’s standard deviation across all items [i.e., (scale-mean_individual_ *minus* all-item-mean_culture_)/all-item-SD_culture_].

### MAIN ANALYSES

Using Latent Gold^®^ 4.5, we tested a model with the standardized experiential ratings as indicators (predictors). **Table 1** provides the results^1^. We examined the Bayesian Information Criterion (BIC; Schwartz, 1978), the Akaike’s Information Criterion (AIC), a variant of the AIC criterion (called AIC_3_, with 3 as penalizing factor) to assess model fit. For all these information criteria (IC), lower number indicates better fit (Magidson and Vermunt, 2004; Nylund et al., 2007). However, we note that simulation studies considering LCA models confirmed that the BIC is the most reliable of the ICs across various model permutations (Lukociene et al., 2010; for an overview, see Fonseca and Cardoso, 2007; Nylund et al., 2007). Additionally, we report entropy, which is measured on a 0–1 scale, with a value of 1 indicating that participants are perfectly classified into latent classes. In general, higher entropy values reflect a more accurate classification and the six-class model had a value of 0.918. The six-class model also possessed the lowest BIC, AIC, and AIC_3_ followed by the five-class model. Additionally, the difference between the BIC models was greater than 2.0, indicating a “positive” difference in fit (Raftery, 1995); hence, we chose the six-class model.

**Table 1:** Latent class analysis statistics.

Number of classes	Log-likelihood	BIC	AIC	AIC_3_	Number of parameters	Composition of best-fitting model
1	-8316.92	16721.47	16661.83	16675.83	14	
2	-7798.52	15778.56	15655.04	15684.04	29	
3	-7614.30	15504.02	15316.59	15360.59	44	
4	-7513.21	15395.73	15144.42	15203.42	59	
5	-7440.25	**15343.70**	**15028.50**	**15102.50**	74	
6	-7390.37	15337.85*	14958.74*	15047.74*	89	*Most-me Class 1* (*n* = 159; 81.8% Most-me): W=39.6%, EA=23.9%, SA=20.1%, SEA=16.4%
						*Least-me Class 1* (*n* = 105; 80.0% Least-me): W=51.4%, EA=15.2%, SA=15.2%, SEA=18.1%
						*Least-me Class 2* (*n* =79; 70.9% Least-me): W=40.5%, EA=25.3%, SA=11.4%, SEA=22.8%
						*Most-me Class 2* (*n* = 67; 95.5% Most-me): W=61.2%, EA=11.9%, SA=3.0%, SEA=23.9%
						*Least-me Class 3* (*n* = 62; 96.8% Least-me): W=64.5%, EA=11.3%, SA=11.3%, SEA=12.9%
						*Converging Class* (*n* = 51; 51.0% Least-me): W=23.5%, EA=31.4%, SA=31.4%, SEA=13.7%
7	-7361.61	15374.21			104	
8	-7325.14	15395.17			119	
9	-7301.33	15441.44			134	
10	-7270.84	15474.35			149	

*Bold denotes the two lowest BIC, AIC, and AIC3 values and the asterisk denotes the lowest BIC, AIC, and AIC3 values in the column. In the right-most column, the identified classes are listed in descending order by size. Furthermore, W refers to Westerners, EA refers to East Asians, SA refers to South Asians, and SEA refers to South-East Asians.*

**FIGURE 1:**
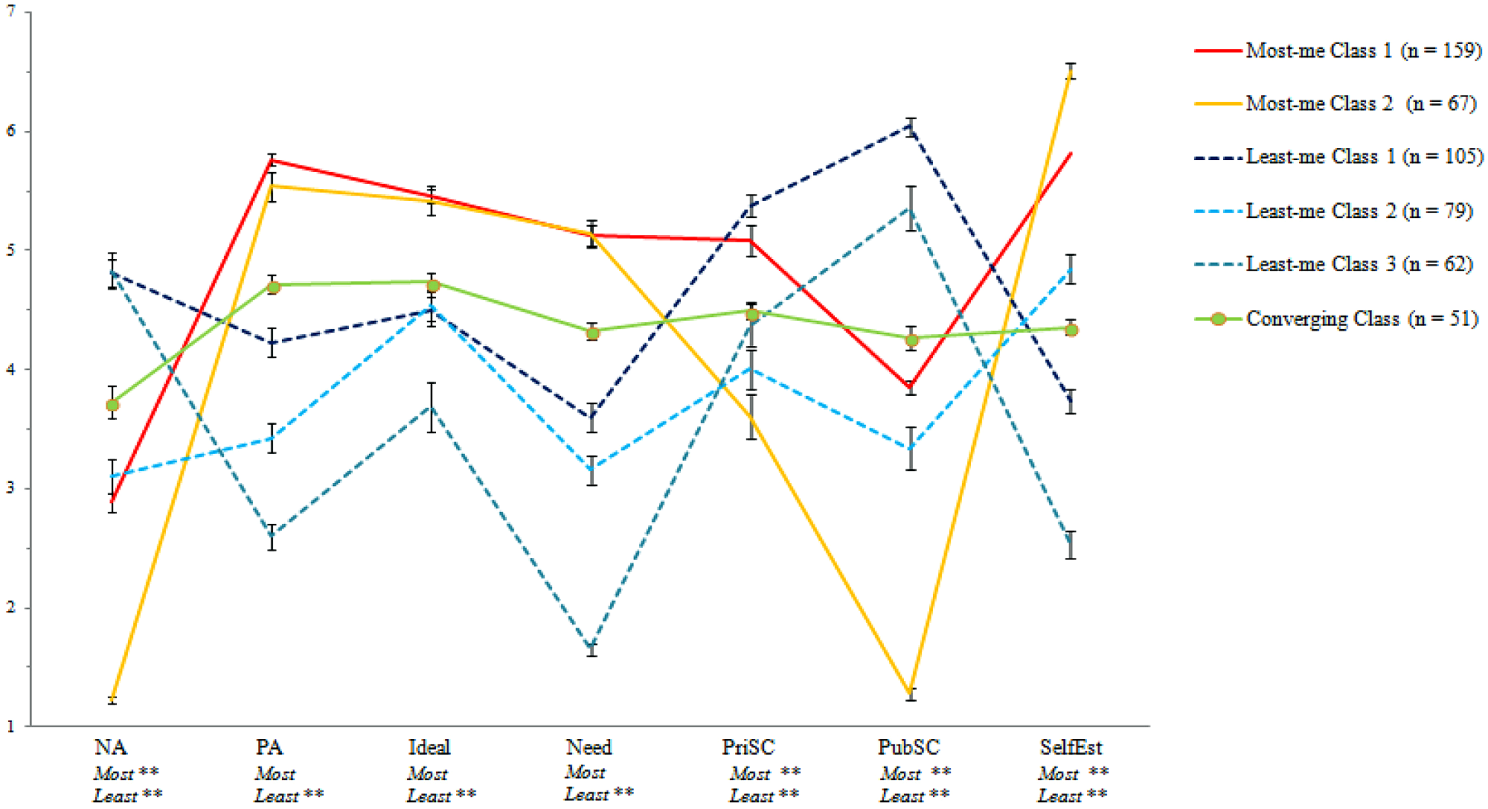
**Six-class experiential profile (means and standard errors)**.

Next, we compared the classes’ experiential profiles (**Figure 1**). Consistent with previous findings, on average, inauthenticity classes were higher in NA, lower in PA, lower in ideal self-overlap, lower in need satisfaction, and lower in self-esteem than authenticity classes; two of three inauthenticity classes were also higher in public self-consciousness than authenticity classes (Lenton et al., 2013a). The converging class generally fell between the authenticity and inauthenticity classes, with scores around each scale’s midpoint. Crucially, significant differences emerged among the two authenticity and three inauthenticity classes.

One-way ANOVAs demonstrated that the three inauthenticity classes differed with respect to all seven indicators (**Figure 1**). *Post hoc* analyses (Bonferroni) revealed that the classes were significantly different from one another at *p <* 0.05 across the seven indicators *except* that Least-me Classes 1 and 3 did not differ in NA, Classes 1 and 2 did not differ in ideal self-overlap, and Classes 2 and 3 did not differ in private self-consciousness.

We label Least-me Class 3 experiences *extraordinary inauthenticity*, as they involved much more NA than PA, very little (if any) need satisfaction and self-esteem, and strong public self-consciousness. Least-me Class 1 also was associated with relatively high NA and, in fact, the strongest public and private self-consciousness, but PA, self-esteem, and need satisfaction were not entirely absent. Thus, we label this class *self-conscious inauthenticity*. Least-me Class 2 was an experientially deflated class: NA and public self-consciousness were not especially high, but neither were PA, need satisfaction, and private self-consciousness. These narratives were associated with a modicum of self-esteem and ideal self-overlap (perhaps due to the higher number of most-me narratives contained in this class), but still lower than either of the most-me experiences. We label this class *deflated inauthenticity*.

The two most-me classes were more similar to one another, as they were associated with equally strong PA, ideal self-overlap, and need satisfaction. Most-me Class 2 (vs. Most-me Class 1) narratives, however, were rated significantly lower on NA and public and private self-consciousness, and higher on self-esteem. That is, these narratives represented very affectively positive events with little public self-consciousness. Hence, we refer to the two sub-classes as *everyday* vs. *extraordinary* forms of authenticity.

Below we provide exemplar narratives from each class to convey the wide variety of situations described by participants.

#### Least-me Class 1 (self-conscious inauthenticity)

[...] I failed to get a scholarship and my father was accused of [rape]. I tried to ignore it and pretend I [was] not affected but it wasn’t the real me. I just [portrayed myself] to be someone who is strong. I focused myself [on] work and never tried to confess to someone except to God.

When I found out I was cheated [on] by my husband, that’s [when] I felt the least me. Because it did not [happen] only once but 3 times. The text in his phone was my evidence of what he did to me.

#### Least-me Class 2 (deflated inauthenticity)

At [some] relatives’ place [...] whom I only meet once a year. I grew up being a shy and quiet boy. However, with my experiences ever since I was 16 years old (I’m 27 now), I have become more outspoken and interactive. Nevertheless, I remain the shy and quiet boy that my relatives know me to be. I can’t become the person I am today, outspoken and interactive, all of sudden around my distant relatives. As such, I remain quiet to them, only speaking when spoken to. This is different from my life where I would usually be the one to start a conversation.

I currently feel the “least” me. I just moved [...and] I have been taken out of time and place. My physical environment is different. [...] My friends aren’t accessible in the physical form like they were in the past. I have to learn how to find my way in this city. I also need a new bank, doctor, dentist, pharmacy, grocery store, apt. utilities, etc. I am going to a new school. I was accustomed to a certain life that I feel that I will have to, to an extent, build up again. This makes me a bit sad because those common routine things are not there anymore. [...]

#### Least-me Class 3 (extraordinary inauthenticity)

[...]Today when I was on my lunch break a girl walking towards me dropped down on her knees and put her hands together. She didn’t say anything but she closed her eyes and put her hands together. She looked like she hadn’t eaten or showered, she also looked like she was high on some kind of drug. I stopped and looked at her and then I walked away and I feel absolutely terrible about it. I can’t get the image out of my mind of the way that she looked and the way that she fell on her knees. I feel like a horrible person and am disgusted with myself that I walked away. [...]

I felt the least like me when involved in an intimate relationship that ticked all the right boxes [...] yet everything felt, wrong, I felt disconnected, I felt fake, I felt (despite my boyfriend’s devotion and attention) that there was something essentially wrong about me/ with me. That I was not who I had thought I was, that I didn’t know who I was, that maybe I never had. [...] I wanted to stop pretending, but I didn’t even know what I was pretending [...] I ended the relationship [...]and strived in a strange dissociated state at building a musical/creative community [...] I was looking for like souls, but I didn’t know what it was about me that was different. Years [passed]...and one day I was out dancing with friends and saw/felt the presence of another across the room. I looked at him and knew he was intersex, and that I was also! Over the past year personal/ family archaeology has revealed the history of hidden difference, and explained the weight of horrified unexplained shame that was projected on me from my family. I have been liberated [...]We close our eyes to what we don’t expect to see - but now my eyes are open and I am free to be me without apology or shame.

#### Most-me Class 1 (everyday authenticity)

This current time in my life I feel I have really reached the real me and found myself. I am living on my own, with my own puppy in which I have done an amazing job training. I live alone, and pay my bills on my own, and go to school. I love having a place of my own, and my own things that I have worked hard for, even if it means not having extra money.

I am bisexual, but not one that is openly out. So whenever I am with [my significant other], I feel that I can be myself. It’s as if there is nothing that she doesn’t know about. We connect on an emotional level wherein sometimes, words are not needed between us. It doesn’t have to be anywhere specific. The fact that I am with her is enough. We laugh at the same things, we enjoy the same movies, we read the same books, or we crave the same food. It’s as if we are two parts of a whole. I know that there is nothing that I can’t tell her, and for that I am grateful.

#### Most-me Class 2 (extraordinary authenticity)

[...] I felt most me when I was 20 years of age ... about year after I had suffered a head injury and had to wear a plastic mask that burn victims wear for 6 months. That experience brought me to a place where I didn’t give a shit what anyone thought of me. I guess to pinpoint an actual event, I would say... I traveled to Europe on my own and traveled for a summer on my own. I talked with everyone and asked everyone questions... I tried and practiced the language of the country I was without concern with if I sounded dumb or not exact. [...] I embraced life.

I was on holiday in Majorca with my parents. I was 19 years old. I remember being so happy to be there, and had such a sense of freedom that I spontaneously burst into song. I sang loudly and proudly for all to hear and was not bothered that everyone was looking at me. I didn’t feel silly, just absolutely delighted to be there. It only lasted a few minutes, but it felt magnificent. I have never felt so happy and natural in all my life!

#### Converging Class

Traveling with a group of friends and without the company of my parents for the first time when I was 21. I could do what I want[ed] without being mindful of my parents’ views.

I’ve moved to California where I have no friends and no family. I feel [...] alone and try to accommodate my significant other because I have no one else in my life. I have never been one to do this.

### DEMOGRAPHIC ANALYSES

To determine whether participants were distributed differently across the classes by their demographics, we conducted a multinomial logistic regression. We entered “Class” as the categorical dependent variable (six levels, with the most populous class – *everyday authenticity* – set as the reference category), with sample (1 vs. 2), gender (male vs. female), age (standardized), and culture (four levels, with the most populous culture, Western, set as the reference category) entered as predictors. Culture [*X*^2^(15) = 42.04, *p* = 0.001] and age [*X*^2^(5) = 13.66, *p* = 0.018] were significant predictors of class assignment, but sample [*X*^2^(15) = 2.76, *p* = 0.737] and gender [*X*^2^(15) = 7.61, *p* = 0.179] were not.

**Table 1** describes the distribution of cultures across classes. We list significant (<0.05) comparisons only. East Asians (vs. Westerners) reported fewer experiences of *extraordinary authenticity* (Odds Ratio or OR = 0.33, 95% CI = 0.13–0.81) and *extraordinary inauthenticity* (OR = 0.25, 95% CI = 0.10–0.64). South Asians (vs. Westerners) also reported fewer experiences of *extraordinary authenticity* (OR = 0.09, 95% CI = 0.02–0.43).

With respect to age, there was one significant effect: the *converging class* comprised significantly younger participants than the *everyday authenticity* class (OR = 0.56, 95% CI = 0.35–0.90).

## DISCUSSION

Authenticity and inauthenticity come in different flavors, but remain discriminable. The LCA showed that, whereas PA, ideal self-overlap, self-esteem, and need satisfaction strongly distinguished authenticity from inauthenticity (these were lower in all three inauthenticity classes than in both authenticity classes), the different flavors of each authenticity and inauthenticity owed more to variability in NA and public and private self-consciousness.

The experiential hallmark of state authenticity is strong PA, ideal self-overlap, need satisfaction, and self-esteem, as both forms of authenticity possessed these attributes (and extraordinary authenticity possessed even higher self-esteem than everyday authenticity). State inauthenticity, in contrast, bore no experiential hallmark, as the three classes differed on all seven indicators. Indeed, the constructs responsible for discriminating authenticity from inauthenticity also helped differentiate among the three inauthenticity classes. Specifically, some forms of inauthenticity do not necessitate low PA, ideal self-overlap, self-esteem, or need satisfaction. This is surprising given the presumed strong link between inauthenticity and negative outcomes (Hochschild, 1983; Goldman and Kernis, 2002; Heppner et al., 2008; Wood et al., 2008). Future work might examine how people buffer mood and self-esteem from certain inauthenticity experiences.

That negative affect strongly differentiated among types of authenticity and inauthenticity is not surprising, given that affect is known to influence the self more generally (Sedikides, 1992). NA was at its nadir in extraordinary authenticity, but strong in extraordinary inauthenticity. Yet, one authenticity class (everyday) was associated with as much negative affect as one inauthenticity class (deflated). Thus, contrary to the implications of prior findings, the relation between negative affect and felt authenticity is not perfectly linear (Heppner et al., 2008; Lenton et al., 2013b).

Public self-consciousness also differed between the authenticity and inauthenticity subtypes. Although the extraordinary forms of each authenticity and inauthenticity represent opposite ends of the public self-consciousness spectrum, not all experiences of authenticity are without public self-consciousness and, conversely, not all experiences of inauthenticity are imbued with public self-consciousness. We speculate that public self-consciousness and negative affect together may have a causal relation with (in)authenticity, as negative affect – especially anxiety – often coincides with public self-consciousness (Carver and Scheier, 1986). That is, it is possible that high public self-consciousness may lead to negative affect, or that their co-presence is sufficient to induce extraordinary inauthenticity. Conversely, when both negative affect and public self-consciousness are distinctly absent, extraordinary authenticity may be enabled. Future research could examine the interplay between public self-consciousness and negative affect in inducing state (in)authenticity, as this study cannot disentangle potential causes from effects.

Additionally, everyday authenticity was associated with moderate public self-consciousness and strong positive affect. Hence, public self-consciousness without concomitant negative affect may not produce the inauthenticity experience. Still further, public self-consciousness and negative affect are not necessary for inauthenticity: Deflated inauthenticity possessed only modest levels of each. Future work might examine the interactive effects of these constructs on state authenticity.

The findings provide support for one of the hypotheses concerning the private self-consciousness–authenticity relation: Everyday authenticity was associated with much higher private self-consciousness than was extraordinary authenticity, and the former was also higher in private self-consciousness than two of the three inauthenticity classes. Stated otherwise, one form of authenticity is indeed associated with introspection and self-examination (Kernis and Goldman, 2006; Wood et al., 2008). Yet, everyday authenticity also was associated with increased public self-consciousness. This pattern suggests that everyday authenticity is more social than extraordinary authenticity and, as such, may correspond to Kernis and Goldman’s (2006) “relational orientation” component of dispositional authenticity. In other words, everyday authenticity may represent the phenomenological experience of reflecting on and disclosing aspects of oneself (good and bad) to others.

The existence of the converging class indicates that the phenomenological experience of authenticity may not always be different from that of inauthenticity. Examination of narratives in the converging class showed that many highlighted contrasting emotions (e.g., an inauthenticity narrative describing the person getting excited when discussing friends’ problems; an authenticity narrative describing the person’s uncertainty regarding their suitability for a new job, but then discovering they were perfectly suited to it). Thus, while deflation (flatter affect) was associated with inauthenticity, truly ambivalent emotions are more difficult to classify. The implications for well-being of these types of (in)authenticity experiences remain to be determined.

We also note that the experiential profile of some instances of authenticity resembled more closely inauthenticity and vice versa (though the latter occurred less frequently). Conspicuously, these “miscategorizations” were less likely to occur for the extraordinary than the non-extraordinary forms of inauthenticity and authenticity (**Table 1**). The extraordinary forms were more “uncontaminated” and, thus, arguably represent the prototypical experience, at least for Westerners. Still, similar to the existence of the converging class, these “miscategorizations” suggest that authenticity and inauthenticity are not necessarily experientially different from one another (at least not on the set of relevant variables we assessed) and, thus, should not be considered opposites.

Although culture plays a role in state authenticity, that role is not all-encompassing (Robinson et al., 2013; Slabu et al., in press). Specifically, all four cultures were represented in every class, but not uniformly so. East Asians and South Asians were less likely to report extraordinary authenticity; East-Asians also reported significantly fewer instances of extraordinary inauthenticity. In contrast, Westerners’ narratives were relatively over-represented in these two classes. Thus, although there was considerable overlap across the cultures, different cultures also possessed somewhat distinct conceptualizations of what “being my true self” feels like.

The cross-cultural results are not the result of our Western participants having a more extreme response style, as we adjusted for this prior to the LCA. These culture effects may instead reflect differences in self-construal (Triandis, 1995). The study from which many of our cross-cultural participants emanated (Slabu et al., in press) found that Singaporeans (who made up most of our South-East Asian sample) were less independent than their counterparts from the US (who made up most of our Western sample), but more independent than their Chinese and Indian counterparts (who made up most of our East-Asian and South-Asian samples, respectively). That is, our South-Asian and East-Asian participants were strongly interdependent and the Westerners strongly independent, with the South-East Asians in between. Valuing individual uniqueness and autonomy over group harmony and achievement may put one at special risk of extraordinary inauthenticity, but may also make one more likely to experience extraordinary authenticity.

The findings may be limited by the retrospective nature of our methods, as reconstructive memory is generally subject to distortion (Loftus and Palmer, 1974). For example, individuals’ enduring goals or motives shape their autobiographical memories (McAdams, 1993; Sedikides and Green, 2009). To the extent that the same is true of our participants’ ratings, the results in part amplify attributes of the “most-me” or “least-me” stories in line with their goal. Future research might examine whether *in situ* experiences of authenticity and inauthenticity also comprise distinct sub-type experiences and if these differ cross-culturally.

An alternative interpretation of these results is that they reflect differences in personality rather than in types of authenticity experiences. Although we cannot rule out this possibility – after all, LCA is more commonly used to differentiate individuals rather than states of being (Nylund et al., 2007; Jung and Wickrama, 2008) – we argue that it is at least equally plausible (if not more so) that the authenticity and inauthenticity classes represent different types of experiences that nearly anyone can have given the “right” circumstances. We know, for example, that state (in)authenticity is separable from trait (in)authenticity. That is, regardless of their level of trait authenticity, individuals experience both authenticity and inauthenticity, and such experiences occur with regularity (Lenton et al., 2013a). Furthermore, we instructed participants to recount a single episode of either authenticity or inauthenticity and to rate that episode on several dimensions; we did not instruct them to describe how they generally experience authenticity or inauthenticity. Stated otherwise, we asked participants to consider and evaluate only a single, specific experience of authenticity (or inauthenticity), not how authenticity (or inauthenticity) feels regardless of its antecedents or context. Finally, there is no reason to believe that these experiential profiles are mutually exclusive, especially in light of the types of situations that participants described. That is, “feeling self-conscious when among strangers at a party” does not preclude that one might experience more extraordinary inauthenticity in a different situation or that would experience either everyday or extraordinary authenticity in still other circumstances as well. Taken together, while some individuals may be more likely than others to report experiences of one or another class of (in)authenticity, the procedures of our studies make it more likely that the classes represent different types of (in)authenticity experiences than of people, and the extant literature reinforces this point.

Overall, the current results demonstrate that (in)authenticity is not phenomenologically unitary, as we uncovered distinct types of experiences. The implications of experientially distinct types of authenticity and inauthenticity for well-being constitute an exciting research avenue. For example, deflated inauthenticity actually may be more deleterious than extraordinary inauthenticity if the former occurs more frequently or is not as easily explained away. Furthermore, this multifaceted view of authenticity may be important in shaping people’s expectations for when they have successfully attained their “true self.” Seeking to experience everyday authenticity or avoid only extraordinary inauthenticity may decrease the perceived difficulty of being one’s true self.

## Conflict of Interest Statement

The authors declare that the research was conducted in the absence of any commercial or financial relationships that could be construed as a potential conflict of interest.

## Footnotes

1 We ran the analysis 10 times to preclude the possibility of identifying a local rather than global solution (Nylund et al., 2007). The values shown in **Table 1** were replicated and thus reflect a stable (global) solution.
